# Afamin Levels and Their Correlation with Oxidative and Lipid Parameters in Non-diabetic, Obese Patients

**DOI:** 10.3390/biom12010116

**Published:** 2022-01-12

**Authors:** Imre Juhász, Szilvia Ujfalusi, Ildikó Seres, Hajnalka Lőrincz, Viktória Evelin Varga, György Paragh, Sándor Somodi, Mariann Harangi, György Paragh

**Affiliations:** 1 Department of Emergency Medicine, Faculty of Medicine, University of Debrecen, 4032 Debrecen, Hungary; juhaszimre86@gmail.com (I.J.); somodi@belklinika.com (S.S.); 2 Doctoral School of Health Sciences, Faculty of Public Health, University of Debrecen, 4032 Debrecen, Hungary; ujfalusisz@gmail.com; 3 Department of Internal Medicine, Faculty of Medicine, University of Debrecen, 4032 Debrecen, Hungary; seres@belklinika.com (I.S.); lorincz_hajnalka@belklinika.com (H.L.); vargavikievelin@gmail.com (V.E.V.); harangi@belklinika.com (M.H.); 4 Department of Dermatology, Roswell Park Comprehensive Cancer Center, Buffalo, NY 14203, USA; gparagh@gmail.com; 5 Department of Cell Stress Biology, Roswell Park Comprehensive Cancer Center, Buffalo, NY 14203, USA

**Keywords:** obesity, insulin resistance, afamin, HDL subfractions, vitamin E

## Abstract

**Background:** Afamin is a liver-produced bioactive protein and features α- and γ-tocopherol binding sites. Afamin levels are elevated in metabolic syndrome and obesity and correlate well with components of metabolic syndrome. Afamin concentrations, correlations between afamin and vitamin E, afamin and lipoprotein subfractions in non-diabetic, obese patients have not been fully examined. **Methods:** Fifty non-diabetic, morbidly obese patients and thirty-two healthy, normal-weight individuals were involved in our study. The afamin concentrations were measured by ELISA. Lipoprotein subfractions were determined with gel electrophoresis. Gas chromatography–mass spectrometry was used to measure α- and γ tocopherol levels. **Results:** Afamin concentrations were significantly higher in the obese patients compared to the healthy control (70.4 ± 12.8 vs. 47.6 ± 8.5 μg/mL, *p* < 0.001). Positive correlations were found between afamin and fasting glucose, HbA1c, hsCRP, triglyceride, and oxidized LDL level, as well as the amount and ratio of small HDL subfractions. Negative correlations were observed between afamin and mean LDL size, as well as the amount and ratio of large HDL subfractions. After multiple regression analysis, HbA1c levels and small HDL turned out to be independent predictors of afamin. **Conclusions:** Afamin may be involved in the development of obesity-related oxidative stress via the development of insulin resistance and not by affecting α- and γ-tocopherol levels.

## 1. Introduction

The incidence of obesity—in line with the incidence of insulin resistance and type 2 diabetes mellitus—is increasing worldwide [1]. The development of abnormal carbohydrate metabolism in obesity is relatively common, but the exact underlying pathomechanism is still not fully understood [2].

In the past few decades, hepatokines were the center of focus as potential causative agents. The characterization of these bioactive, liver-derived proteins may help us better understand obesity-associated non-communicable diseases [3]. One such glycoprotein is afamin, a member of the albumin family, discovered in 1994 [4]. Afamin is mainly produced by the liver, but high concentrations can also be found in human ovarian follicles [5] and cerebrospinal fluid [6]. It features specific α- and γ-tocopherol binding sites and thus may play a crucial role in vitamin E metabolism [7]. Increased weight, as well as higher lipid and glucose levels have been observed in transgenic mice carrying the human afamin gene [8]. Afamin levels are elevated in metabolic syndrome and obesity, and correlate well with the components of metabolic syndrome (waist circumference, body mass index—BMI, triglyceride—Tg, glucose, low-density lipoprotein-cholesterol—LDL-C) in obesity and type 2 diabetes mellitus [8]. Similar results have been found in patients with polycystic ovary syndrome (PCOS), a condition often accompanied by metabolic syndrome [9]. In a large multi-center study—involving more than 20,000 patients—significant correlations were observed between the incidence and prevalence of type 2 diabetes and afamin levels [10]. A recent study suggested afamin as a potential novel biomarker of increased hepatic lipid content in prediabetes and type 2 diabetes [11].

The key event in the pathomechanism of the metabolic syndrome is the development of insulin resistance resulting in hyperinsulinemia. Elevated insulin levels enhance fatty acid uptake in the liver and, therefore, endogenous very low-density lipoprotein (VLDL) production is increased [12]. Insulin resistance is also responsible for the decreased activity of lipoprotein lipase, an enzyme responsible for the breakdown of triglyceride-rich lipoproteins [13]. Increased production combined with decreased elimination results in further elevation in Tg levels [14]. There is an inverse relationship between Tg elevation and high-density lipoprotein (HDL) cholesterol levels [15]. The reason for this is the altered maturation of HDL particles in the presence of lipid components released during the breakdown of Tg [16]. The Tg content of HDL increases and the metabolism of these particles by hepatic lipase accelerates [13,16]. There is also a change in the composition of LDL: the level of small, dense LDL particles—prone to oxidation—increases, resulting in elevated oxidized LDL (oxLDL) concentrations and accelerated atherogenesis [17].

Hypertriglyceridemia, hyperglycemia, hyperinsulinemia, elevated small, and dense LDL and low HDL levels together result in endothelial dysfunction and promote atherosclerosis [18]. According to previous studies, 13% of afamin is bound to lipoproteins in circulation, partly to HDL (containing A1 apolipoprotein) and preferably to small, dense HDL particles [6]. In total, 97% of lipid-soluble α- and γ-tocopherol is bound to lipoproteins in plasma [19]. However, afamin is not the only protein capable of specifically binding these tocopherols [20]. Although the antioxidant potential of α- and γ-tocopherol is not unequivocally proven in clinical studies, tocopherol could prevent oxidative alterations in LDL via the inhibition of lipid peroxidation [21]. In previous studies, plasma afamin levels were measured in patients suffering from secondary complications of already abnormal carbohydrate and lipid levels [10,11,22]. Furthermore, we previously observed moderately decreased circulating afamin concentrations—in line with HDL-C and ApoA1 levels—after LDL apheresis [23]. Therefore, our goal in this study was to measure afamin levels in morbidly obese patients without abnormal carbohydrate parameters. We expected elevated afamin concentrations in these patients and strong correlations between lipid parameters (especially with HDL and LDL subfractions), the amount of oxidized LDL, α- and γ-tocopherol levels, and afamin.

## 2. Materials and Methods

### 2.1. Patients

A total of 50 metabolically healthy, obese (BMI > 30 kg/m^2^) patients and 32 normal-weight, healthy, age- and sex-adjusted control patients were enrolled in our study. Anthropometric data and laboratory parameters are summarized in Table 1. Patients with liver, kidney, endocrine disorders (including both type I and II diabetes mellitus) or malignancies were excluded. Exclusion criteria also included pregnancy, breast feeding, smoking and regular alcohol consumption. All subjects gave their informed consent for inclusion before they participated in the study. The study was conducted in accordance with the Declaration of Helsinki, and the protocol was approved by the Ethics Committee of University of Debrecen and the Medical Research Council (registration number: DE RKEB/IKEB 5513B-2020 and ETT TUKEB IV/7989-1/2020/EKU, respectively).

### 2.2. Sample Collection and Laboratory Measurements

Venous blood samples were obtained from both obese and control patients after 12 h of fasting. A Cobas c501 (Roche Ltd., Mannheim, Germany) analyzer was used to measure carbohydrate and lipid parameters. Oral glucose tolerance test (OGTT) was performed in order to exclude diabetes mellitus in obese patients (75 g of glucose). HbA1c and insulin levels were also measured at the same time. The widely used formula was used to calculate homeostasis model assessment-estimated insulin resistance (HOMA-IR) values (fasting insulin concentration (µU/mL) × fasting glucose concentration (mmol/L)/22.5). Serum samples were separated by centrifugation at 4 °C at 3500× *g* for 10 min. Routine laboratory parameters were determined from fresh sera with a Cobas c501 analyzer (Roche Ltd. Mannheim, Germany). Total cholesterol levels were measured by using enzymatic, colorimetric tests (cholesterol oxidase-p-aminophenazone—GPOD-PAP; Modular P-800 analyzer; Roche/Hitachi). HDL cholesterol and LDL cholesterol levels were determined by a homogenous enzymatic, colorimetric assay (Roche HDL-C plus third generation and Roche LDL-C plus second generation, respectively). Immunoturbidimetric assays (Tina-quant apolipoprotein A-I ver. 2 and Tina-quant apolipoprotein B ver. 2, respectively) were used to measure Apo A-I and ApoB levels. All the tests were performed according to the recommendations of the manufacturer. Sera were kept frozen at −70 °C for subsequent lipoprotein subfraction analysis and ELISA measurements.

### 2.3. Determination of Serum Afamin Level

Serum afamin concentrations were measured by a commercially available ELISA kit (Afamin Human ELISA, cat. number: RD194428100R, BioVendor, Asheville, NC, USA), according to the recommendations of the manufacturer. The intra- and inter-assay variation coefficients were <3.61% and <3.4%, respectively.

### 2.4. Measurement of Serum Oxidized LDL Concentration

Serum concentrations of oxidized LDL (oxLDL) were detected by a commercially available solid phase two-site enzyme immunoassay (ELISA) kit (Mercodia AB, Uppsala, Sweden). Measurements of oxLDL levels in the sera were performed according to the recommendations of the manufacturer. The intra- and inter-assay coefficients of variations were 5.5–7.3% and 4.0–6.2%, respectively, and the sensitivity was <1 mU/L.

### 2.5. Measurement of Serum α- and γ-Tocopherol Levels by Gas Chromatography-Mass Spectrometry

Plasma α- and γ-tocopherol determination was based on the modified method described by Zerbianti et al. [24]. Internal standard method was used for the preparation of standard curves. Methanolic stock solutions of tocopherols (Sigma-Aldrich, St. Louis, MO, USA) were used to prepare dilution series with methanol (Sigma-Aldrich, St. Louis, MO, USA) for α-tocopherol (3.13–25 µg/mL) and for γ-tocopherol (0.063–0.5 µg/mL). Fixed amount (0.4 µg) of methanolic 2,2,5,7,8-Pentamethyl-6-chromanol (Sigma-Aldrich St. Louis, MO, USA) internal standard solution was added to standard solutions. Next, these standards were dried under nitrogen flow and were derivatized with 130 µL Sylon™ HTP sylilating solution (Sigma-Aldrich, St. Louis, MO, USA) for 30 min at 60 °C. After that, standards were dried once again under nitrogen flow and then dissolved in 50 µL n-hexane (Merck, Darmstadt, Germany). For sample preparation, 5 µL of plasma was mixed with 100 µL 2,2,5,7,8-Pentamethyl-6-chromanol (4 µg/mL in methanol) as the internal standard, 100 µL methanol and 95 µL saline. A total of 1 mL n-hexane was added for extraction. Samples were vortexed then centrifuged (3500 rpm, 5 min, room temperature). The uppermost layer was transferred into glass vials and dried under nitrogen flow. During the next phase samples were derivatized the same way as standards described above. Gas chromatography-mass spectrometry measurements were performed with Finnigan Trace GC Ultra connected to Polaris Q mass spectrometer (Thermo Fisher Scientific, Waltham, MA, USA). Samples were injected manually into Agilent J&W column (DB-5MS UI; 60 m × 0.25 m × 0.25 µm) using helium as carrier gas (1 mL/min, constant flow). In total, 2 µL of the previously prepared samples were injected in splitless mode and injector temperature was held at 260 °C throughout our analysis. Initial oven temperature (150 °C) was held for 2 min, then raised to 300 °C with a rate of 25 °C/min, and maintained for further 15 min, giving a total run time of 23 min. The mass spectrometer was operated in selective ion monitoring mode and the chosen ions were: α-tocopherol-TMS 237.3 m/z, γ-tocopherol-TMS 488.4 m/z, 2,2,5,7,8-Pentamethyl-6-chromanol-TMS 292.3 m/z.

### 2.6. HDL Subfraction Analysis

HDL subfractions were measured by using an electrophoretic method on polyacrylamide gel with the Lipoprint System (Quantimetrix Corp., Redondo Beach, CA, USA), according to the manufacturer’s instructions. Lipoprint separates HDL subfractions from human serum based on their size applying preloaded gel tubes for HDL determinations. In total, 25 μL serum was added to the polyacrylamide gel tubes along with 300 μL loading gel solution. Tubes contained Sudan Black as lipophilic dye and were photopolimerized at room temperature for 30 min. Electrophoresis with tubes containing sera samples and the manufacturer’s quality controls were performed at a constant of 3 mA/tube for 50 min. Subfraction bands were scanned with an ArtixScan M1 digital scanner (Microtek International Inc., Redondo Beach, CA, USA) and were identified by their mobility. Ten HDL subfractions were differentiated between VLDL + LDL and albumin peaks, and were grouped into three major classes: large, intermediate and small HDL subfractions. The percentage of large (HDL1-3), intermediate (HDL4-7) and small (HDL8-10) HDL subfractions were analyzed with Lipoware software (Quantimetrix Corp., Redondo Beach, CA, USA). Cholesterol concentrations of HDL particle subsets were calculated by multiplying HDL-C concentrations of samples by the relative area under the curve (AUC) of subfraction bands.

### 2.7. LDL Subfraction Analysis

LDL subfractions were also detected using the Lipoprint System (Quantimetrix Corp., Redondo Beach, CA, USA) according to the instructions of the manufacturer. A total of 25 µL sera was added to polyacrylamide gel tubes along with 200 µL Sudan Black as a lipophilic dye. The sample loading gel mixture was photo-polymerized for 30 min at 20–22 °C prior to electrophoresis at a constant of 3 mA/tube for 60 min.

LDL subfractions were identified by their electrophoretic mobility using VLDL as the starting and HDL as the ending reference point. AUC% for the VLDL, Midband A, B, C (comprising primarily IDL), up to seven LDL subfraction and HDL peaks were calculated by Lipoware computer software (Quantimetrix Corp., Redondo Beach, CA, USA). Percentage of large LDL (large LDL%) was defined as the sum of the percentage of LDL1 and LDL2, whereas percentage of small LDL (small-dense LDL%) was defined as the sum of LDL3-LDL7. Cholesterol concentrations of LDL subfractions were determined by multiplying the relative AUC of subfractions by total cholesterol concentration of the sample. Calculated total LDL-C is comprised of the sum of the cholesterol in Midbands (A, B, C) and LDL subfractions (LDL1-LDL7); and strongly correlates with the directly measured LDL-C. Mean LDL size was calculated by Lipoware software (Quantimetrix Corp., Redondo Beach, CA, USA).

### 2.8. Statistical Analysis

The STATISTICA (ver: 8.0; StatSoft Inc., Tulsa, OK, USA) program was used to analyze data. The Kolmogorov–Smirnov test was used to analyze the distribution of our results. In case of normal distribution, the two sample Student T-test, whereas in case of non-normal distribution the Mann–Whitney u test was performed. Parameters with normal distribution are given as mean ± standard deviation (SD). Other parameters with non-normal distribution are given as median with lower and upper quartiles. Pearson’s correlation was used to analyze the relationship between continuous variables. Multivariate analysis (backward-stepwise method) was used to find those parameters that correlated with afamin levels the most. Results were considered significant if *p* value was <0.05.

## 3. Results

**Figure 1:**
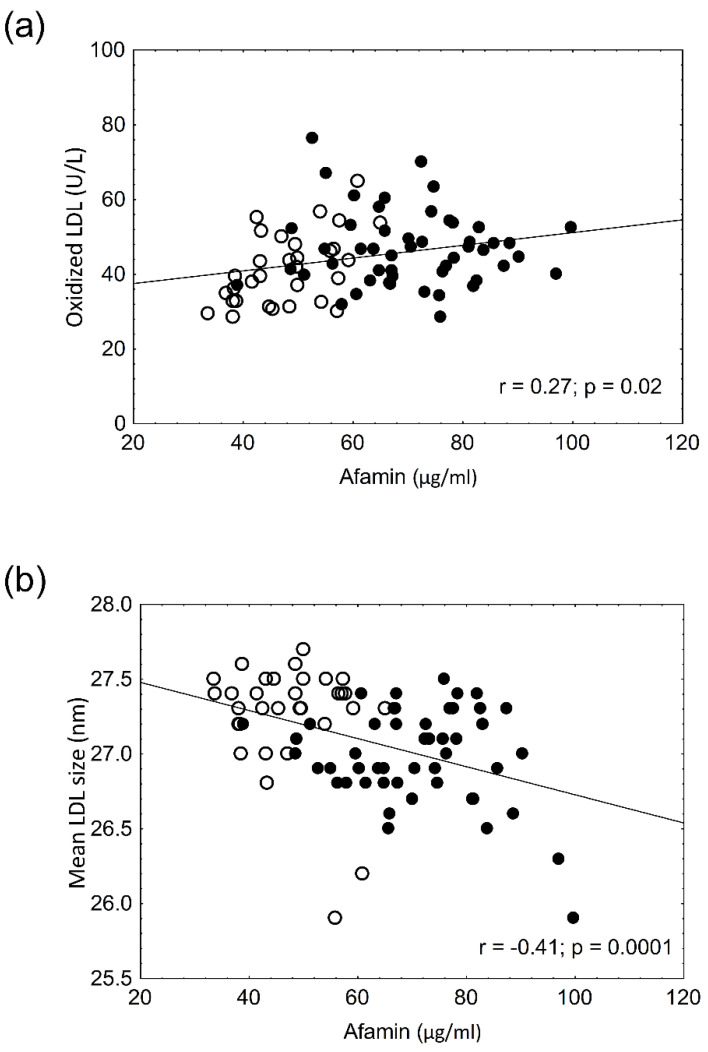
Correlations between afamin and oxidized LDL levels (**a**) and between afamin levels and mean LDL size (**b**) in the study populations (● obese non-diabetic patients vs. ○ lean controls).

**Figure 2:**
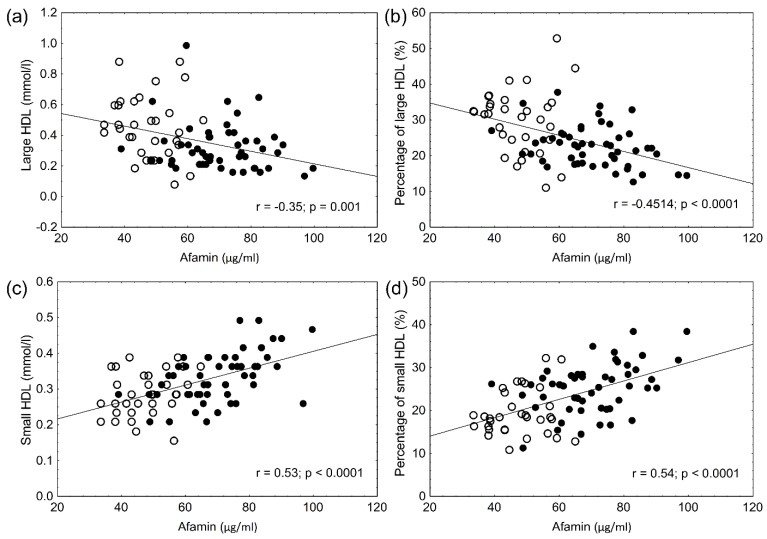
Correlations between concentration of afamin and the level (**a**) and percentage (**b)** of large HDL subpopulation and between concentration of afamin and level (**c**) and percentage (**d**) of small HDL subpopulation in the study participants (● obese non-diabetic patients vs. ○ lean controls).

The anthropometric data and laboratory parameters are summarized in Table 1. The lipid and carbohydrate values significantly differed between obese and control patients; however, these parameters were still in the normal range. Elevated hsCRP indicated low-grade systemic inflammation in obese patients. Uric acid, HbA1c, and fasting glucose levels were significantly higher in obese, non-diabetic patients; however, even these parameters were in the normal range. Two-hour OGTT and HOMA-IR values were used to exclude diabetes (Table 1). The percentage and amount of LDL and HDL subfractions are shown in Table 2. The total HDL concentrations were significantly lower in obese, non-diabetic patients (Table 1). There was also a shift in the HDL particles to small, dense HDL subfractions. The percentage and absolute amount of large HDL particles were significantly lower in obese patients. The percentage and absolute amount of small HDL were significantly higher in obese patients. There was a mild, but significant, decrease in the amount of intermediate HDL (Table 2). The total LDL concentrations—as well as LDL subfractions—were higher in obese patients. The percentage and amount of both large and small LDL subfractions were higher in obese patients. The mean size of LDL particles was significantly smaller in obese patients. Serum afamin concentrations were significantly higher in obese, non-diabetic patients (Table 3). The oxidized LDL levels were higher in obese patients. The α- and γ tocopherol levels were also significantly higher in obese, non-diabetic patients. This difference was still present after α- and γ tocopherol levels were normalized to cholesterol values (Table 3). As previously published in different articles, significant positive correlations were found between BMI (r = 0.582, *p* < 0.001), waist circumference (r = 0.434, *p* < 0.005), glucose (r = 0.468, *p* < 0.001), HbA1c (r = 0.661, *p* < 0.001), uric acid (r = 0.573, *p* < 0.001), hsCRP (r = 0.537, *p* < 0.001), and afamin levels. A significant positive correlation between afamin and oxLDL levels and a significant negative correlation between afamin and mean LDL size were seen (Figure 1a,b). However, the α- and γ-tocopherol levels did not correlate with the afamin concentrations in obese patients (r = 0.20; *p* = 0.2 and r = 0.22; *p* = 0.1, respectively). There were significant negative correlations between the percentage and absolute amount of large HDL and afamin levels (Figure 2a,b) and significant positive correlations between the percentage and amount of small HDL and afamin concentrations (Figure 2c,d). A multiple regression analysis was performed to determine which parameter (age, BMI, waist circumference, glucose, HbA1c, α- and γ-tocopherol, α-tocopherol/cholesterol, γ-tocopherol/cholesterol, oxLDL, the amount and percentage of large and small HDL) affects afamin levels the most. The afamin levels were mainly influenced by waist circumference (β = 0.685, *p* < 0.001), HbA1c (β = 0.291, *p* < 0.01) and the amount of small HDL subfraction (β = 0.282, *p* < 0.05).

## 4. Discussion

To our knowledge, this is the first report on elevated afamin levels in morbidly obese, non-diabetic patients. Afamin levels were 48.1% higher in obese, non-diabetics compared to control patients. This elevation is similar to that observed in diabetics compared to healthy individuals; however, due to methodical differences, the values are not comparable [10]. Lipid and glucose levels were significantly higher in obese non-diabetic patients; however, they were still in the normal range. Despite this, BMI, waist circumference, fasting glucose, Tg, hsCRP and LDL-C levels did correlate well with afamin concentrations in non-diabetic obese patients, similar to diabetics. This suggests that afamin may contribute to the development of insulin resistance at an early stage. Previous studies did not examine the relationship between afamin levels and lipid subfractions in obese patients. We found significant correlations between the percentage and amount of HDL subfractions and afamin levels. The binding of afamin to small, dense HDL subfractions and the shift to these HDL fractions in obesity have been described previously [6,25]. After the multiple regression analysis, the amount of small HDL subfraction turned out to be one major determinant of afamin. The strong correlation observed between HDL subfractions and afamin levels is a remarkable finding and may help us to better understand the function of HDL subfractions.

It is well known that several antiatherogenic proteins—e.g., human paraoxonase-1—are also bound to small, dense HDL subfractions [26,27]. However, the increased amount and percentage of small, dense HDL subfractions alone—as small HDL levels were only 18.1% higher in obese non-diabetic patients—does not explain the significant increase in afamin levels. The role of fat-soluble, herbal molecules, frequently referred to as vitamin E, has been studied for decades [28]. It is widely established that free radicals—produced during increased oxidative stress in inflammatory conditions—cause lipid peroxidation and thus contribute to atherosclerosis [29]. The antioxidant properties of α- and γ-tocopherols—the two most common and widely consumed vitamin E components—have been well described [30,31]. At the same time, results from studies focusing on the effects of vitamin E on atherosclerosis are incomprehensive due to different characteristics and cardiovascular risk between study populations [32,33,34]. Currently, we think that vitamin E supplementation could exert beneficial effects on atherosclerosis in some high-risk patients [35]. The levels of α- and γ-tocopherol were slightly, but significantly, higher in obese, non-diabetic patients. The α- and γ-tocopherol/total cholesterol ratios were also significantly higher in obese patients. Our study was the first to examine α- and γ-tocopherol levels in obese, non-diabetic patients, although the relationship between vitamin E and lipid levels has been previously described in various patient groups [36,37,38]. We did not find any correlation between α- and γ-tocopherol and afamin levels. This suggests that afamin—despite the fact that it features specific α- and γ-tocopherol binding sites—does not play a crucial role in the regulation of vitamin E levels. As oxidized LDL levels are higher in obese, non-diabetic patients, increased α- and γ-tocopherol concentrations could be beneficial in reducing endogen oxidative stress in these patients. However, further studies are needed to evaluate this hypothesis.

Our goal was to better understand the role of afamin, the regulation of lipoprotein metabolism, as well as impaired oxidative stress status in morbidly obese, non-diabetic subjects in order to define the effect of obesity on these parameters without the concomitant effect of carbohydrate disturbances. This information could be useful to estimate obesity-associated cardiovascular risk and may help us to come up with personalized therapeutic strategies in obese subjects.

The limitations of our study must be acknowledged. We possess no detailed information on the eating habits and vitamin E consumption of our patients; however, routine supplementation of vitamin E in this population is uncommon. The data are also limited due to the small number of patients involved. Based on our results, it is not clear whether increased afamin levels in obesity contribute to the development of insulin resistance or are simply unrelated consequences of obesity. Thus, further examinations on larger number of obese individuals and enrolment of prediabetic and type 2 diabetic patients may help to enhance the statistical power of the study. Despite the relatively small number of participants, our findings may underline the potential role of circulating afamin in impaired lipoprotein metabolism in obese and lean non-diabetic subjects.

## 5. Conclusions

Afamin levels were elevated in obese, non-diabetic patients and concentrations did not correlate with α- and γ-tocopherol levels. Strong correlations were found between large and small HDL subfraction levels, oxLDL, mean LDL size, the components of metabolic syndrome and serum afamin concentrations in obese non-diabetics. Elevated concentrations of afamin and their association with lipoprotein subfractions might be useful when assessing obesity-associated cardiovascular risk. Based on our findings, afamin may play a role in the development of early carbohydrate and lipid abnormalities and oxidative stress in obese patients, while afamin levels did not significantly affect α- and γ-tocopherol levels. Further studies are needed to clarify the role of afamin in obesity and the development of insulin resistance.

## Figures and Tables

**Table 1:** Characteristics and laboratory parameters in obese patients and controls.

	Obese Patients	Controls	*p*-Value
	*n* = 50	*n* = 32	
**Gender (F/M)**	43/7	27/5	n.s.
**Age (yrs)**	44.2 ± 13.5	41.8 ± 6.0	n.s.
**BMI (kg/m^2^)**	42.0 ± 8.6	24.2 ± 2.5	<0.001
**Waist circumference (cm)**	119.8 ± 16.9	83.6 ± 9.3	<0.001
**hsCRP (mg/L)**	8.2 (3.2–13.1)	1.4 (0.5–2.5)	<0.001
**sTSH (mU/L)**	1.98 ± 0.98	2.06 ± 1.22	n.s.
**AST (U/L)**	23.5 ± 9.0	18.7 ± 3.9	<0.01
**ALT (U/L)**	29.4 ± 15.3	18.1 ± 7.9	<0.001
**γ-GTP (U/L)**	33.6 ± 21.4	24.3 ± 15.4	<0.05
**LDH (U/L)**	355.6 ± 84	222.2± 73.2	<0.001
**Uric acid (µmol/L)**	315.2 ± 91.6	254.5 ± 63.7	<0.001
**Cholesterol (mmol/L)**	5.0 ± 0.8	5.0 ± 0.8	n.s.
**Triglyceride (mmol/L)**	1.4 (1.1–2.0)	1.0 (0.75–1.39)	<0.01
**HDL-C (mmol/L)**	1.4 ± 0.3	1.6 ± 0.5	<0.001
**LDL-C (mmol/L)**	3.2 ± 0.7	2.9 ± 0.6	<0.05
**ApoA-I (g/L)**	1.48 ± 0.24	1.71 ± 0.31	<0.001
**ApoB (g/L)**	0.86 ± 0.20	0.88 ± 0.23	n.s.
**Glucose (mmol/L)**	5.4 ± 0.7	4.8 ± 0.5	<0.001
**OGTT 0′**	4.9 ± 0.8		
**OGTT 120′**	7 ± 2		
**HbA1C (%)**	5.8 ± 0.5	5.1 ± 0.3	<0.001
**Insulin (mU/L)**	21 ± 15.9		
**HOMA-IR**	3.75 (2.4–6.52)		
**C-peptide (pmol/L)**	1325 (1055–1619)		

Data are presented as mean ± standard deviation (SD) or median (lower-upper quartile). n.s.; non-significant.

**Table 2:** HDL and LDL subfraction distribution and levels in obese patients and controls.

		Obese	Control	*p*-Value
**HDL**	Large HDL%	22.5 ± 5.7	30.9 ± 9.4	<0.05
	Intermediate HDL%	52.3 ± 3.4	50.2 ± 4.7	n.s.
	Small HDL%	25.2 ± 5.9	18.9 ± 5.7	<0.05
	Large HDL (mmol/L)	0.32 ± 0.16	0.53 ± 0.31	<0.05
	Intermediate HDL (mmol/L)	0.71 ± 0.17	0.78 ± 0.17	n.s.
	Small HDL (mmol/L)	0.33 ± 0.07	0.28 ± 0.06	<0.01
**LDL**	Large LDL %	25.8 ± 4.1	21.4 ± 5.9	<0.05
	Small-dense LDL %	2.0 ± 1.6	1.0 ± 2.1	<0.001
	Large LDL (mmol/L)	1.32 ± 0.36	1.08 ± 0.35	<0.01
	Small-dense LDL (mmol/L)	0.11 ± 0.12	0.05 ± 0.11	<0.05
	Mean LDL size (nm) (minimum-maximum)	26.98 ± 0.3 (25.9–27.5)	27.25 ± 0.3 (25.9–27.7)	<0.001

Data are presented as mean ± standard deviation (SD). n.s.; non-significant.

**Table 3:** Afamin, α- and γ tocopherol levels and oxidized LDL in obese patients and controls.

	Obese	Control	*p*
**Afamin (µg/mL)**	70.4 ± 12.9	47.6 ± 8.5	<0.001
**α-tocopherol (µg/mL)**	9.4 (7.9–13.2)	8.2 (7.2–9.7)	<0.05
**γ-tocopherol (µg/mL)**	0.2 (0.16–0.31)	0.12 (0.1–0.17)	<0.001
**α-Tocopherol/cholesterol**	1.95 (1.62–2.51)	1.64 (1.49–1.946)	<0.05
**γ-Tocopherol/cholesterol**	0.04 (0.03–0.06)	0.03 (0.02–0.033)	<0.001
**oxLDL (U/L)**	46.8 ± 10	40.2 ± 10.1	<0.005

Data are presented as mean ± standard deviation (SD) or median (lower-upper quartile).

## Data Availability

All data generated or analyzed during this study are included in this published article. All data generated or analyzed during the current study are available from the corresponding author on reasonable request.
